# Both cis and trans-acting genetic factors drive somatic instability in female carriers of the *FMR1* premutation

**DOI:** 10.1038/s41598-022-14183-0

**Published:** 2022-06-21

**Authors:** Ye Hyun Hwang, Bruce Eliot Hayward, Marwa Zafarullah, Jay Kumar, Blythe Durbin Johnson, Peter Holmans, Karen Usdin, Flora Tassone

**Affiliations:** 1 Department of Biochemistry and Molecular Medicine, University of California Davis, School of Medicine, Sacramento, CA 95817 USA; 2 Laboratory of Molecular and Cellular Biology, National Institute of Diabetes, Digestive and Kidney Diseases, National Institutes of Health, Bethesda, MD USA; 3 Department of Public Health Sciences, University of California, Davis, School of Medicine, Sacramento, CA 95817 USA; 4 Medical Research Council Centre for Neuropsychiatric Genetics and Genomics, Division of Psychological Medicine and Clinical Neurology, School of Medicine, Cardiff University, Cardiff, UK; 5 MIND Institute, University of California Davis Medical Center, Sacramento, CA 95817 USA

**Keywords:** Neurodevelopmental disorders, Genetics

## Abstract

The fragile X mental retardation (*FMR1*) gene contains an expansion-prone CGG repeat within its 5′ UTR. Alleles with 55–200 repeats are known as premutation (PM) alleles and confer risk for one or more of the *FMR1* premutation (PM) disorders that include Fragile X-associated Tremor/Ataxia Syndrome (FXTAS), Fragile X-associated Primary Ovarian Insufficiency (FXPOI), and Fragile X-Associated Neuropsychiatric Disorders (FXAND). PM alleles expand on intergenerational transmission, with the children of PM mothers being at risk of inheriting alleles with > 200 CGG repeats (full mutation FM) alleles) and thus developing Fragile X Syndrome (FXS). PM alleles can be somatically unstable. This can lead to individuals being mosaic for multiple size alleles. Here, we describe a detailed evaluation of somatic mosaicism in a large cohort of female PM carriers and show that 94% display some evidence of somatic instability with the presence of a series of expanded alleles that differ from the next allele by a single repeat unit. Using two different metrics for instability that we have developed, we show that, as with intergenerational instability, there is a direct relationship between the extent of somatic expansion and the number of CGG repeats in the originally inherited allele and an inverse relationship with the number of AGG interruptions. Expansions are progressive as evidenced by a positive correlation with age and by examination of blood samples from the same individual taken at different time points. Our data also suggests the existence of other genetic or environmental factors that affect the extent of somatic expansion. Importantly, the analysis of candidate single nucleotide polymorphisms (SNPs) suggests that two DNA repair factors, *FAN1* and *MSH3*, may be modifiers of somatic expansion risk in the PM population as observed in other repeat expansion disorders.

## Introduction

Over 35 different inherited genetic disorders are caused by the expansion of a specific short tandem repeat tract^1^. In these repeat expansion disorders, the repeat is unstable showing a strong expansion bias. The *FMR1* disorders or Fragile X-related disorders (FXDs), are members of this group that result from the presence of an evolutionarily conserved, but expansion-prone, CGG repeat tract at the 5′ end of the transcriptional unit of the X-linked *FMR1* gene. The repeats are situated upstream of the open reading frame for FMRP, an RNA binding protein important for the regulation of translation in post-synaptic neurons in response to synaptic activation. The repeats are thought to modulate mGluR-dependent enhancement of FMRP synthesis via non-AUG initiated (RAN) translation through the repeat tract^2^. Premutation alleles (PM) have 55–200 repeats and are associated with a risk of developing one or more of the PM associated disorders, Fragile X-associated tremor/ataxia syndrome (FXTAS), Fragile X-associated primary ovarian insufficiency (FXPOI), and Fragile X-associated neuropsychiatric disorders (FXAND)^3–6^. Pathology is thought to arise from some deleterious effect of the excess number of repeats in the *FMR1* transcript^7^. Carriers of PM alleles are also at risk of transmitting larger alleles to their children, with increasing CGG repeat number being associated with increased risk^8^. In particular, female PM carriers with ~ 90 CGG repeats, have a > 90% probability of transmitting alleles with > 200 CGG repeats to their children. Such alleles are known as full mutation (FM) alleles and result in Fragile X syndrome (FXS), a neurodevelopmental disorder that is the most common inherited form of intellectual disability and the most common monogenic cause of autism spectrum disorder. Pathology in this instance is thought to be related to the repeat-mediated silencing of the *FMR1* promoter^9^. The prevalence of the PM allele among the general population is 1:110–200 females and 1:430 males. However, the PM disorders have a variable penetrance with 40–75% of males and 8–16% of females developing FXTAS^10,11^ and ~ 20% of females developing FXPOI^12,13^.

The increased use of higher resolution techniques for the analysis of PM alleles has demonstrated that some carriers of PM alleles show somatic repeat size mosaicism, i.e., the presence of two or more alleles of different sizes in a particular tissue. Previous studies of mosaicism have focused on individuals containing a combination of multiple discrete alleles often in both the PM and FM range^14–23^. The origin of the smaller alleles is uncertain, but likely reflects contractions of larger alleles. The second type of mosaicism is also present in PM carriers, in which multiple alleles differing by a single repeat are seen in some individuals^24^. This form of mosaicism is reminiscent of the products of somatic expansion seen in an FXD mouse model and in humans with other repeat expansion diseases^25^. Molecular modeling of these products suggests that they arise via small but frequent events that accumulate over the lifetime of the individual^26^. In an FXD mouse model, the frequency with which these events occur differs between tissues and cell types. While this phenomenon has not been extensively examined in the *FMR1* disorders, it has been reported to occur in humans for other repeat expansion diseases such as Huntington's Disease (HD) and Myotonic Dystrophy type 1 (DM1)^27–29^. The extent of this somatic expansion has been shown to be affected by repeat length and purity as well as a variety of genetic factors with the extent of expansion affecting the age of onset and severity of many of these diseases^29–39^. This study represents the first study of the somatic instability of the *FMR1* repeat in a large cohort of female PM carriers.

## Materials and methods

### Study

Peripheral blood was collected from a total of 426 PM female participants after signing an informed consent form and using a protocol approved by the UC Davis Institutional Review Board.

For the analysis of the correlation of a subset of molecular measures, data from the entire cohort of 426 females were used. For the analysis of the correlation between instability and molecular measures, data from a subset consisting of 384 participants was used. Some individuals were excluded from this subset because the quality of the capillary electrophoresis trace was too poor to allow calculation of instability (n = 19), no AR value was available (n = 1), or the allele corresponding to that on the inactive X could not be identified (n = 8). Individuals with an activation ratio (AR, defined as the percentage of cells carrying the normal allele on the active X chromosome) of > 0.8 (n = 14) who showed no evidence of expansion were also excluded since in these individuals the proportion of alleles able to expand would be relatively small and thus any expansion, should it occur, would be difficult to detect.

For the study of changes in premutation allele stability over time, a subset of 24 female PM participants was selected, based on the availability of at least two blood draws taken a minimum of 2 years apart (mean 6.7; SD 2.9). The age mean was 46.7 (SD 19.5); the mean of the CGG repeats (based on the draw at the first visit) was 100.1 (SD 27.2) (Supplementary Table 1).

### CGG sizing, methylation status, AGG interruptions, and SNP selection

Genomic DNA (gDNA) was isolated from 3 ml of peripheral blood by using the Gentra Puregene Blood Kit (Qiagen, Valencia, CA, United States). CGG repeat allele size and methylation status were assessed using a combination of PCR and Southern Blot analysis. A PCR that specifically targeted *FMR1* amplification (AmplideX PCR/CE, Asuragen, Inc.) was used to determine CGG repeat length and PCR products were visualized by CE and analyzed as previously reported^40^. Southern blotting was performed using the Stb12.3 *FMR1* specific chemiluminescent intronic probe, as detailed in Ref.^41^. Briefly, 10 μg of isolated gDNA was digested with *EcoRI* and *NruI*, run on an agarose gel, transferred to a nylon membrane, and hybridized with the *FMR1*-specific dig-labeled StB12.3. Southern Blot analysis was also used to determine the methylation status of the *FMR1* alleles (Activation ratio, AR, and the percent of methylation) as previously described^42^.

To visualize the methylation status of alleles by capillary electrophoresis a modified version of the assay described in Ref.^43^ was employed. Briefly, 600 ng of genomic DNA was placed in a 40 μl volume of 50 mM Tris.HCl pH 9.0, 1.75 mM MgCl_2,_ 22 mM (NH_4_)_2_SO_4,_ and 1 μl of *HindIII* restriction enzyme were added. This was divided into two equal aliquots and 0.5 μl of *HpaII* restriction enzyme was added to one. Digestion was allowed to proceed overnight at 37 °C. 5 μl of each digest was then made to 20 μl containing 50 mM Tris–HCl pH 9.0, 1.75 mM MgCl_2,_ 22 mM (NH_4_)_2_SO_4_, 2.5 M betaine, 2% DMSO, 0.5 μM each primer, 0.2 mM dATP and dTTP, 0.475 mM dCTP and dGTP, and 0.75U of KAPA2G Robust HotStart polymerase. The PCR conditions were 98 °C for 3 min, 32 cycles of 98 °C for 30 s, 65 °C for 30 s, and 72 °C for 210 s, followed by 72 °C for 10 min. The primers used are:

Not FraxC: AGTTCAGCGGCCGCGCTCAGCTCCGTTTCGGTTTCACTTCCGGT.

Not FraxR4: FAM-CAAGTCGCGGCCGCCTTGTAGAAAGCGCCATTGGAGCCCCGCA.

The number of AGG interruptions was determined by using a triplet primed PCR protocol as described in Ref.^8^, visualized by CE, and analyzed with Gene Mapper software. The number of AGG interruptions in a sample was determined based on the number of sharp depressions visualized by capillary electrophoresis (CE) images^8^.

A total of ten single nucleotide polymorphisms (SNPs) were investigated in a subset of 384 PM female participants for whom the extent of somatic instability could be reliably determined. The choice of SNPs was based on their significant association with instability in other trinucleotide repeat expansion disorders^38^. SNP analysis was performed using the Taqman Single Nucleotide Polymorphism Allele Discrimination Assay for sample genotyping (Applied Biosystems, Inc., Foster City, CA). Predesigned TaqMan assays were used for genotyping. Briefly, probes were mixed with TaqMan Master Mix in a ratio of 2.5 TaqMan Master Mix to 0.125 µl of SNP probe per well, and aliquoted into plates containing 50–100 ng of genomic DNA. A visualization of the cluster plots was performed for each plate to ensure the absence of poor clustering of the SNP. Internal positive and negative controls with all the known genotypes for each SNP were included in each plate. Genotypes were determined using Applied Biosystems automated Taqman genotyping software, SDS v2.1. Genotype data were blind for statistical analysis.

### *FMR1* mRNA expression levels

Total RNA was isolated from 2.5 ml of peripheral blood collected in PAXgene Blood RNA tubes using the PAXgene Blood RNA Kit (Qiagen, Valencia, CA, United States) and quantified using the Agilent 2100 Bioanalyzer system. RNA isolation was performed in a clean and RNA-designated area. cDNA was synthesized as previously described^44^. *FMR1* transcript levels were measured by performing reverse transcription followed by real-time PCRs (qRT-PCR). qRT-PCR was performed using both Assays-On-Demand from Applied Biosystems (Applied Biosystems, Foster City, CA, United States) and custom-designed TaqMan primers and probe assays^44^.

### Measurement of instability

Two different metrics for the extent degree of expansion were used. Since the expansion is limited to the active X chromosome, the smaller alleles represented by Peak 1 represent the originally inherited allele. Our primary measure of expansion, ∆Rpts, is the difference in the number of repeats in a repeat profile between the modal expanded allele (Peak 2) and modal stable allele (Peak 1). Since in males X inactivation does not occur, we adapted a second metric from Ref.^26^ which is based on the increase in the dispersion of the allele populations in the PCR profile. This was calculated by first identifying the modal peaks of the stable (Peak 1) and unstable (Peak 2) allele populations. The RFU values of the peaks exceeding a threshold value (≥ 0.2 × RFU of modal peak) in each population were then converted into a histogram which was treated as being derived from a normal distribution and the standard deviation of that distribution became the dispersion (D) value. To minimize the contribution of alleles in Peak 1 to the dispersion of Peak 2 (D2) and vice versa, we determined the dispersion metric of Peak 2 (D2) by using only Peak 2 and peaks lying to the right of it. Similarly, the dispersion of Peak 1 (D1) was calculated by using only Peak 1 and peaks lying to the left of it.

To determine the proportion of alleles that expand, both the area under the stable peaks in a PCR profile (StableArea) and the area under the curve of the unstable peaks (UnstableArea) were calculated. The proportion of alleles that expand (AUC2) is given by UnstableArea/ (UnstableArea + StableArea) and the proportion of alleles that are stable (AUC1) is then 1 − AUC2.

### Statistical analysis

Statistical analysis was used to determine the correlation between the *FMR1* molecular measures, instability, age, CGG repeat size, AGG interruption, *FMR1* mRNA, and AR. *FMR1* mRNA expression was analyzed by CGG repeat number using linear regression, adjusting for activation ratio (AR) by including this as a covariate. The largest CGG repeat number was used for subjects with different numbers of CGG repeats reported. The above analyses were conducted in R version 4.0.5 (2021-03-31). The overall correlation of factors with instability (as measured by Peak2 − Peak1) was determined using the CORR Procedure, along with the generation of Pearson correlation coefficients. Relationships of individual factors with instability were determined through GLM Procedure. Association of repeat expansion with genetic and other risk factors was tested by negative binomial regression, using the glm.nb () function in R. We estimated the variance inflation factors for each variable in R using the VIF() function in the ‘regclass’ package. The VIFs ranged from 1.13 (AGG) to 2.97 (Peak1), which are comfortably below the cutoff of 5 commonly used to indicate problematic collinearity^45^.

## Results

### Study participants

Blood samples were collected from a total of 426 female PM carriers. The studies and all protocols were carried out in accordance with the Institutional Review Board at the University of California, Davis. All participants gave written informed consent before participating in the study in line with the Declaration of Helsinki. Capillary electrophoresis PCR profiles were determined for the PM alleles in everyone as previously described^40^. Standard practice is to report the number of repeats present in the most common allele as the individual’s repeat number. The number of AGG interruptions was determined by triplet-primed PCR as previously described^8^. The activation ratio (AR), the fraction of normal alleles that are located on the active X chromosome was determined by Southern blot analysis^42^. The *FMR1* mRNA levels were determined by real-time PCR as described previously^44^. The ages of the participants in this study at the time their blood was drawn, their CGG repeat number, number of AGG interruptions, AR, and *FMR1* mRNA levels are shown in Table 1.

**Table 1:** Molecular measures of the 426 and of the subset of 384 female PM carrier groups.

Molecular measures	Total Group, n = 426			Subset, n = 384			Unstable, n = 361	Stable, n = 23	Welch Two Sample t-test	Actual value
	n	Mean	Std. Dev	n	Mean	Std. Dev	Mean ± Std. Err	Mean ± Std. Err		
CGG repeat	425**	92.08	22.94	383**	90.40	19.00	91.69 ± 0.98	70.13 ± 2.65	< 0.0001	~ 3e−8
AGG	426	0.75	0.78	384	0.78	0.79	0.72 ± 0.04	1.65 ± 0.12	< 0.0001	~ 6e−8
AR	424	0.54	0.17	382	0.53	0.16	0.53 ± 0.008	0.58 ± 0.04	0.135153	
*FMR1* mRNA	401	2.18	0.91	361	2.21	0.89	2.24 ± 0.05	1.60 ± 0.18	0.001696	
Age	423	42.49	17.18	381	42.01	16.84	42.80 ± 0.87	29.76 ± 3.99	0.003882	
AUC1	413	0.71	0.23	384	0.71	0.21	0.69 ± 0.01	0.99 ± 0.007	< 0.0001	~ 8e−60
AUC2	413	0.29	0.23	384	0.29	0.21	0.31 ± 0.01	0.01 ± 0.007	< 0.0001	~ 9e−60
D1	412	1.41	0.61	384	1.36	0.32	1.40 ± 0.02	0.77 ± 0.007	< 0.0001	~ 4e−120
D2	411	2.44	2.09	383	2.50	2.01	2.66 ± 0.10	0 ± 0	< 0.0001	~ 2e−83
**AGG**										
0		197 (46.2%)*			171 (44.5%)*		170 (47.1%)*	1 (4.3%)*		
1		138 (32.4%)*			127 (33.1%)*		121 (33.5%)*	6 (26.1%)*		
2		91 (21.4%)*			86 (22.4%)*		70 (19.4%)*	16 (69.6%)*		

*Percentage of females relative to the total number, presenting with 0, 1 or 2 AGG interruptions.

**Number of females for whom the CGG repeat allele size was included (one participants was removed as she was a double heterozygous- two premutation alleles).

### Characterization of somatic expansion

**Figure 1:**
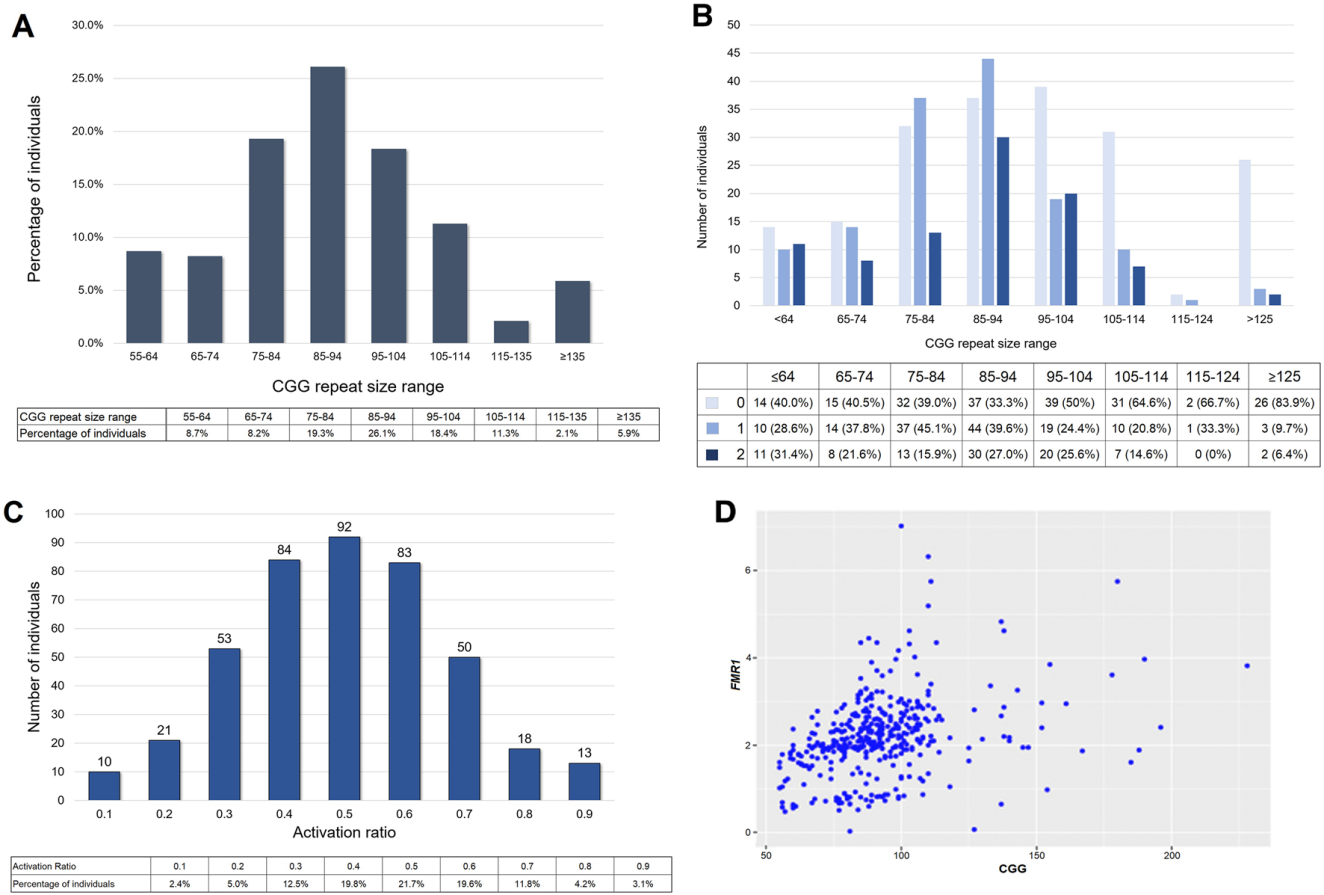
Molecular measures of the study population (n = 426). (**A**) Distribution of CGG repeat numbers in the premutation allele. (**B**) Distribution of AGG interruptions by allele size. A number of individuals and the percentage of individuals are indicated per each category (0, 1, or 2 AGG interruptions within the premutation allele). (**C**) Activation Ratio (AR) distribution in the female participants (n = 424). (**D**) Correlation of CGG repeat number and *FMR1* mRNA level after correction for AR (n = 401, p < 0.0001).

The CGG repeat number showed a normal distribution in our study population (Fig. 1A). The proportion of alleles with no interruptions increased from 40% for alleles with ≤ 64 repeats to > 80% for alleles with ≥ 125 repeats (Fig. 1B). The AR for the study participants was also normally distributed with a mean of ~ 0.5 (Fig. 1C), as previously reported^46^. There was no significant association of repeat size with AR. Consistent with previous reports, higher levels of *FMR1* mRNA were associated with larger repeat lengths (Fig. 1D) even after correction for AR p < 0.0001.

**Figure 2:**
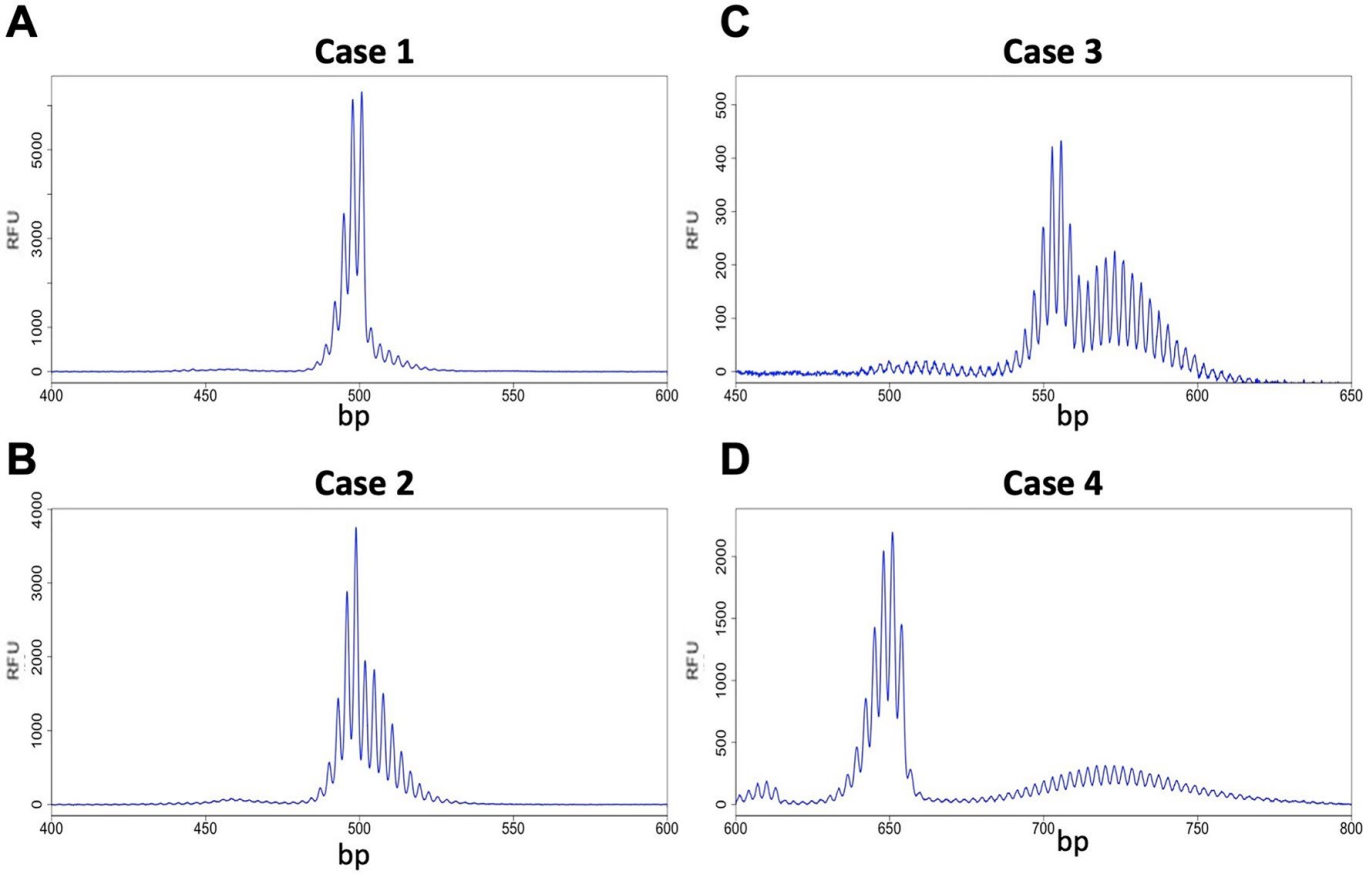
Examples of different types of PCR profiles observed in female PM carriers. Capillary electrophoretograms for 4 different females focusing on the PM allele size range showing the profiles for a stable allele (case 1) and three alleles with increasing levels of somatic expansion.

A variety of different repeat PCR profiles were seen. Some females showed a single sharp and asymmetric PCR profile with a small number of PCR products smaller than the modal allele (Fig. 2A). This is like the PCR profile seen in the blood of very young female PM mice or in the tissue of mice with mutations that block somatic expansion^47,48^. As such, this PCR profile likely reflects a stable allele population with little, or no, somatic expansion, and with some, if not all, of the peaks smaller than the modal allele representing PCR “stutter”. Other individuals showed PCR profiles in which a “shoulder” was seen corresponding to alleles larger than the modal allele (Fig. 2B). The third group of women had a clear bimodal distribution of allele populations with the smaller allele population showing a narrow distribution of allele sizes and the larger allele population showing a broader distribution (Fig. 2C,D). These profiles are like those seen in older female PM mice with a genetic background permissive to somatic expansion. In mice, the smaller of the two allele populations in older animals is similar in size to the alleles present in the tail at 3 weeks of age, an approximate measure of the number of repeats in the originally inherited allele, and the size of this population does not change over time. In contrast, the larger of the two allele populations tend to have a modal repeat number that increases with the age of the animal and thus reflects alleles that have expanded or gained repeats during the animal's lifetime^49^.

**Figure 3:**
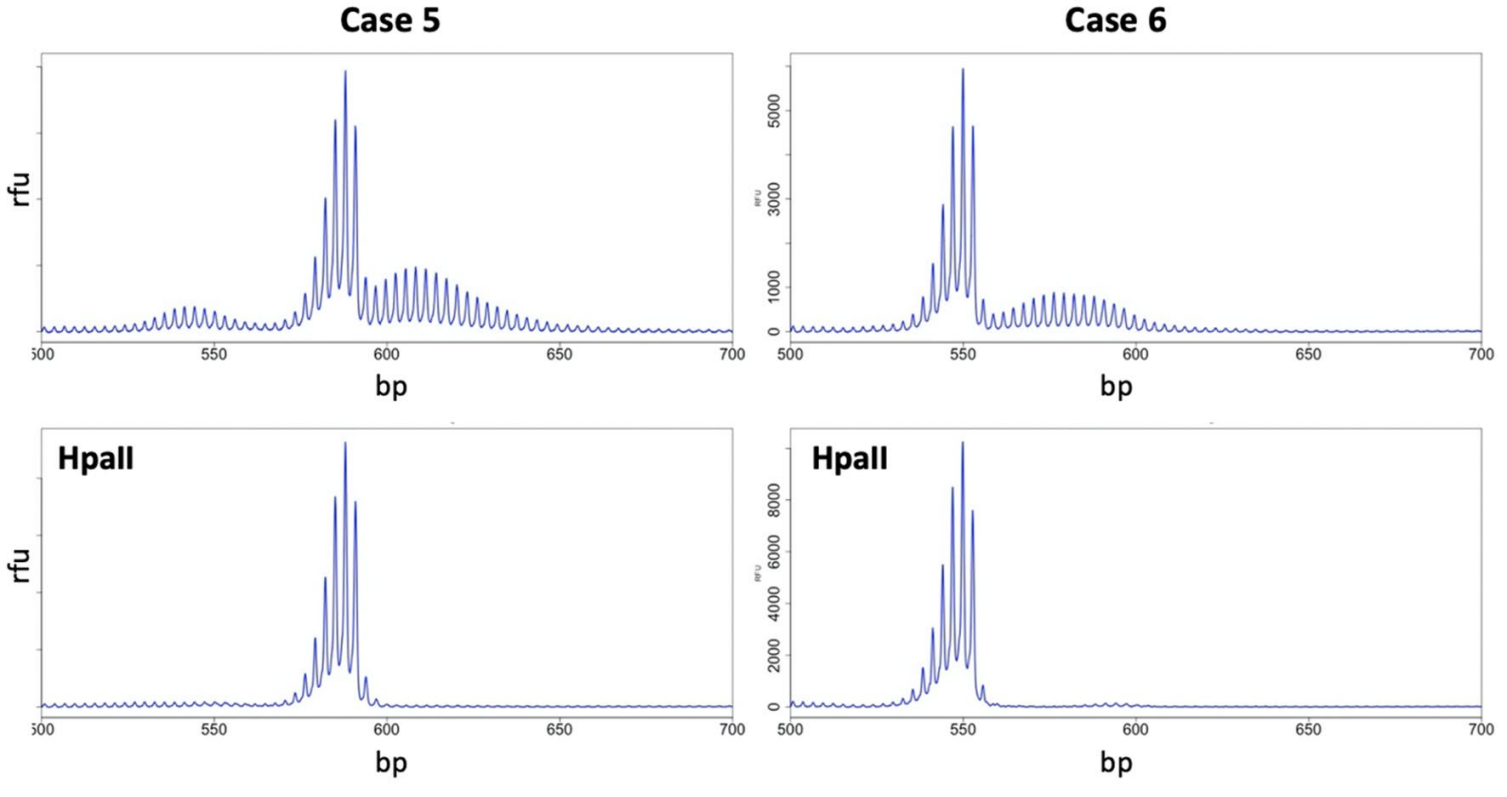
Expansion is limited to PM alleles on the active X chromosome. PCR profiles of two PM female carriers without (top panels) and with (bottom panels) *HpaII* digestion show the loss of the larger of the two allele populations on predigestion of the template with *HpaII* which preferentially eliminates alleles on the active X chromosome.

Interestingly, as in mice, *HpaII* pre-digestion of the PCR template from women with evidence of alleles larger than the modal allele eliminates such alleles from the PCR profile resulting in the production of a unimodal PCR profile characteristic of stable alleles (Fig. 3). Since *HpaII* is a methylation-sensitive enzyme with recognition sites within the amplicon used for PCR analysis of the repeat, pre-digestion eliminates any PCR template derived from an active X chromosome. Thus, the disappearance of these products after *HpaII* digestion suggests that they are derived from the active X chromosome. We interpret this to mean that these products represent expanded alleles with expansions being limited to the active X as in mice.

**Figure 4:**
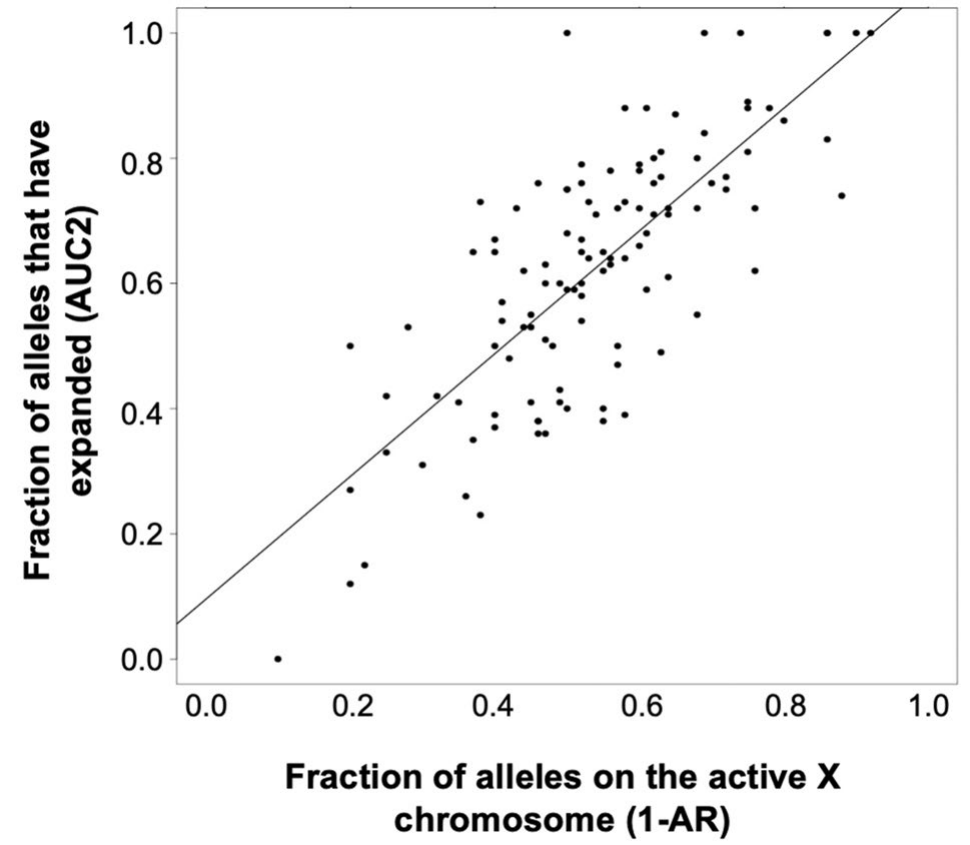
Correlation between the percentage of PM alleles on the active X chromosome and the fraction of alleles that have expanded.

The association between expansion and the presence of the PM allele on the active X is supported by the fact that there is a direct relationship between the fraction of alleles that expand, as assessed by an estimation of the area under the curve of the expanded allele (AUC2) and the fraction of alleles where the PM is on the active X (1 − AR) (Fig. 4). Thus, the allele population with the smaller repeat number corresponds to unexpanded alleles on the inactive X, with the modal repeat number likely reflecting the repeat number present on the originally inherited allele. This is consistent with our previous more limited analysis^49^ and suggests that expansions are limited to the active X chromosome, as they are in mice^47^. This indicates that transcription or a euchromatin configuration is required for these expansions.

**Figure 5:**
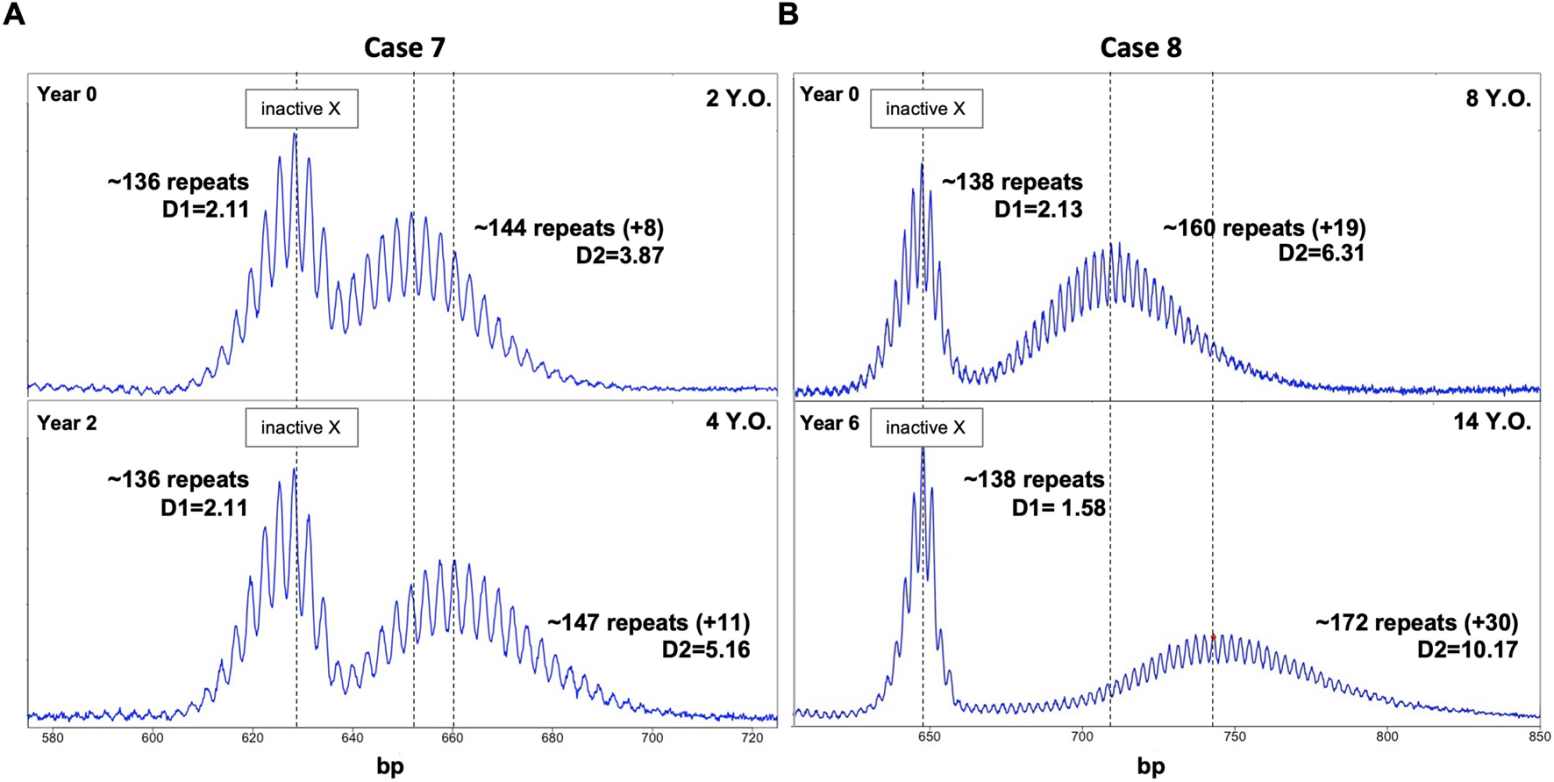
CE analysis shows the changes in the extent of somatic expansion over time in peripheral blood cells from two female PM carriers. The X-axis indicates the number of base pairs, and the Y-axis indicates relative fluorescence intensity.

To investigate the PM allele stability over time, a subset of 24 female participants with specimens available from multiple blood draws, was selected. In 20 of the cases examined, the time between draws was < 10 years. Eight participants showed changes in CGG repeat number (1–12 CGGs; Supplementary Table 1 and Fig. 5). The remaining sixteen individuals (66.7%) showed no evidence of change in their repeat PCR profile between draws, regardless of the age at first sampling and the time between draws. Of these, 11 had < 96 CGG repeats and five had alleles > 96 CGG, with three of the alleles > 96 repeats having AGG interruptions. The other eight individuals showed evidence of a change in the PCR profile with an increase in the modal number of CGG repeats seen in the larger of the two allele populations. Seven of these individuals had inherited alleles with > 96 CGG repeats and no AGG interruptions. A female with ~ 144 CGG repeats in her expanded allele at the first blood draw at two years of age, showed an allele representing a gain of ~ 8 repeats relative to her originally inherited allele (Fig. 5A). She had alleles with a mean repeat number of ~ 147 CGG repeats at the second draw two years later i.e., the gain of three repeats in 2 years (Fig. 5B) shows the PCR profile of a female with ~ 160 CGG repeats on her expanded allele at the first blood draw at eight years of age, 19 repeats more than the original allele. At the second blood draw six years later, the expanded alleles had gained an average of an additional 11 CGG repeats. In addition, as we previously described in an FXD mouse model^24^, the size distribution of expanded alleles broadens with age. This is consistent with mathematical modeling which suggests that each expansion event adds one-to-two repeats^26^. As a result, over time the dispersion of the population of expanding alleles, D2, increases.

### Relationship between the extent of expansion, AR, AGG, age, and the dispersion of the expanded alleles.

The fact that the smaller of the two alleles corresponds to the originally inherited allele and the larger corresponds to those that have expanded would suggest that the difference in the modal number of repeats of the expanded and stable peaks, a metric we call ∆Rpts, reflects the extent of somatic expansion. We used this metric to examine the relationship between the extent of expansion and AGG number, AR, and age. For this purpose, we excluded alleles with AR > 0.8 that showed no evidence of expansion on the grounds that the absence of a detectable second peak might reflect expansions present at levels below the limit of detection by capillary electrophoresis, as could occur if extensive expansion had happened.

In addition, we excluded poor quality capillary electrophoresis traces and individuals where the stable peak could not be identified leaving us with 384 individuals. We then used ∆Rpts as a measure of expansion and performed negative binomial regression of this on the initial repeat number, AGG, AR, age, and the fraction of stable vs unstable alleles (represented by the area under the curve (AUC) of peak 1 and peak 2). We found a significant association between ∆Rpts and the size of the original allele along with a significant direct relationship with age (Table 2).

**Table 2:** Relationship of ∆Rpts to D1, D2, and other molecular measures.

	Estimate	Std. Error	p-value
D1	− 0.06	0.17	0.74
D2	0.34	0.02	< 2e−16
Peak 1	0.02	0.004	1.40E−07
AGG	− 0.23	0.06	0.0001
Age	0.01	0.003	1.19E−05
AR	0.49	0.28	0.08
Amount of FMR1	0.000412	0.05	0.99

The values in this table refer to a multivariable negative binomial regression of ∆Rpts on all of the molecular measures simultaneously.

**Figure 6:**
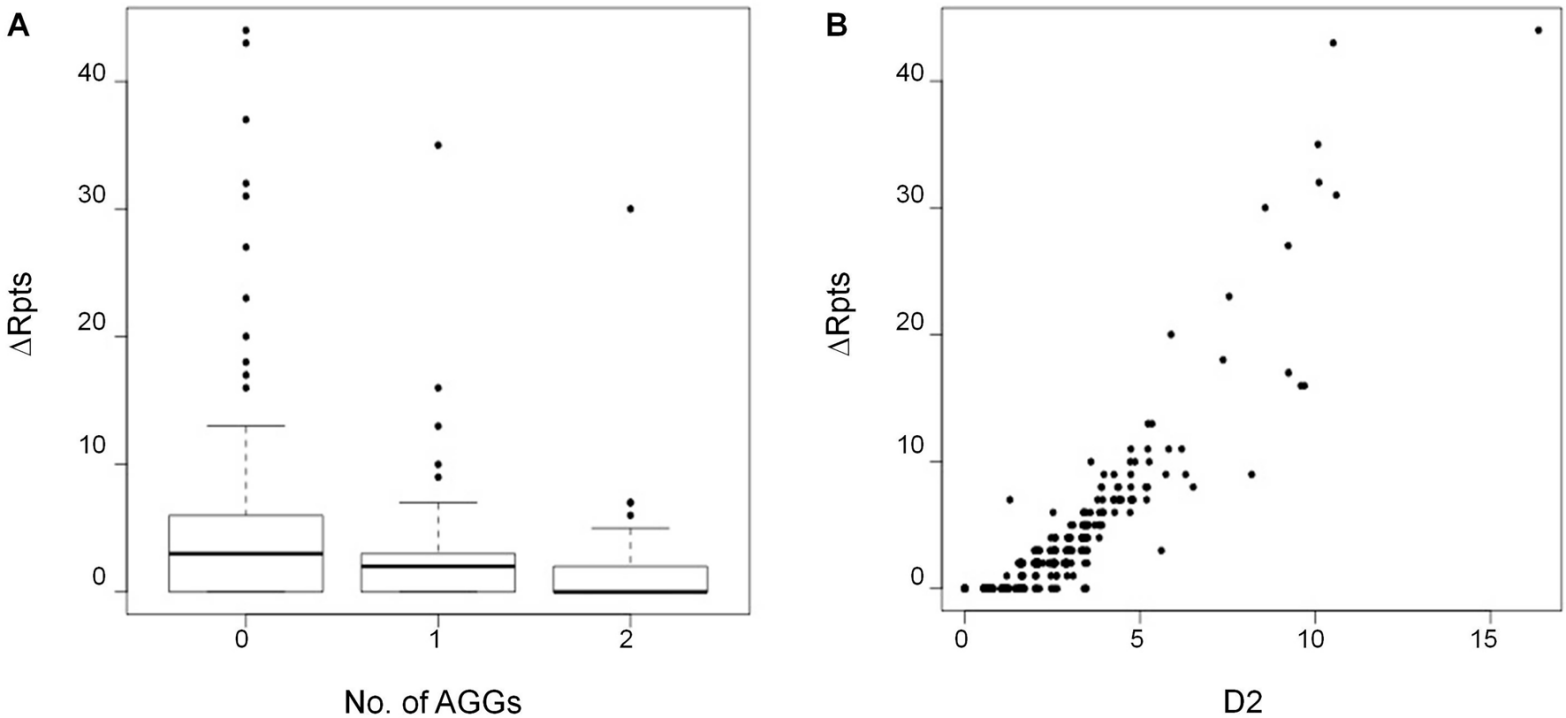
Relationship between instability (∆Rpts) and the number of AGG interruptions (**A**) or the D2 metric (**B**) showing the inverse relationship between ∆Rpts and the number of AGG interruptions and the direct relationship with D2.

There is also an inverse relationship between ∆Rpts and the number of AGG interruptions (Fig. 6A) which is consistent with the stabilizing effect of AGGs observed on intergenerational transmission^50,51^. Since the dispersion about the mean of the expanding alleles increases with increasing expansion, we also tested the association of the ∆Rpts metric with a measure of the dispersion of the stable (D1) and unstable alleles (D2). There was a significant association between the ∆Rpts metric and D2 (Table 2). This is consistent with the data shown in (Fig. 6B) in which the heterogeneity of the expanding allele population increases with time. There was no association with D1 consistent with the fact that the size distribution of the stable allele population shows no increase over time. There was also no relationship between instability and the amount of *FMR1* transcript after correction for the initial repeat number, AGG, AR, and age.

### Genetic factors affecting the expansion

Genome-Wide Association Studies (GWAS) have identified a number of single nucleotide polymorphisms (SNPs) that are significantly associated with the risk of somatic expansion or age of disease onset in various other Repeat Expansion Diseases^29–38^. To assess whether some of the same SNPs were associated with somatic expansion risk in our PM population, we examined the association of the ∆Rpts metric with 10 single nucleotide polymorphisms (SNPs) previously found to be associated with a variation in the age of onset, disease severity or extent of somatic expansion in studies of other Repeat Expansion Diseases. Of the selected ten SNPs chosen and reported in Table 3, two, rs701383 and rs150393409, showed a significant association with the extent of instability, although neither of them would survive correction for multiple testing.

**Table 3:** Correlation between SNPs associated with different repair genes and allele instability.

SNP	Candidate modifier gene(s) (and distance in kb)	Test allele	Effect (Std. Error)	p-value	HW p-val
rs1650742	*MSH3 (0), DHFR (40.1)*	T	0.12 (0.08)	0.12	0.37
rs1799977	*MLH1 (0)*	G	− 0.03 (0.08)	0.73	0.93
rs274883	*LIG1 (0)*	G	− 0.001 (0.09)	0.99	0.31
rs34017474	*FAN1 (0), MTMR10 (0)*	T	− 0.02 (0.07)	0.78	0.8
rs35811129	*FAN1 (6.04) & MTMR10 (0)*	G	0.02 (0.08)	0.78	0.38
rs3791767	*PMS1 (8.8)*	C	− 0.07 (0.09)	0.4	0.06
rs701383	*DHFR (8.77), MSH3 (37.2)*	G	− 0.22 (0.08)	0.007	0.86
rs74302792	*PMS2 (31.3)*	T	− 0.05 (0.11)	0.61	0.81
rs145821638	*LIG1 (0)*	C	− 0.11 (0.53)	0.84	0.94
rs150393409	*FAN1 (0)*	G	− 0.70 (0.31)	0.02	0.74

## Discussion

In this study, we describe the first large-scale characterization of somatic expansion in female premutation allele carriers. We show that most PM carriers show some degree of somatic expansion in blood as evidenced by their PCR profile and by the serial sampling of a subset of individuals. The extent of this expansion is related to the CGG-repeat number and inversely related to the number of AGG interruptions as with intergenerational expansions^14,46,52^. There was also a relationship between the extent of expansion and age, consistent with the observation of a maternal age effect on the risk of a female PM carrier having a child with an FM allele^49–51^. We also showed that the extent of expansion correlates with the proportion of the PM allele that is on the active X chromosome (Fig. 3). This is consistent with the fact that expansion in humans requires transcription or open chromatin as it does in mice^47^. While expansions were not seen on the inactive X chromosome, we observed a relationship between AR and the extent of expansion of the allele on the active X. No evidence of CGG repeat allele contractions was seen in this data set, although the occurrence of low-frequency contraction events or contraction events that generate heterogenous deletion products cannot be definitively excluded.

The measurement of somatic expansion in females is facilitated by the fact that expansion is limited to alleles on the active X chromosome and thus that the size of the inherited allele can be inferred from the size of the allele on the inactive X. However, this is not possible in males. Our demonstration that the extent of expansion as measured by ∆Rpts shows a direct relationship with DM2, the dispersion of the expanded allele about the mean, suggests that the DM metric could be useful for examining somatic expansion in male PM carriers.

The demonstration of the association of the rs701383 SNP with the extent of somatic expansion is of interest since this SNP has located 8.77 kb from the dihydrofolate reductase (*DHFR*) gene and 37.2 kb from *MSH3*, whose gene product is important for mismatch repair and is required for both somatic and germline expansion in the mouse model of FXDs^1^. rs701383 is an eQTL for *MSH3* in GTEx, that is significant in several tissues (minimum p = 1.5 × 10^–71^ in cultured fibroblasts) with the minor allele (A) at rs701383 being associated with higher expression of MSH3^32^. rs701383 is an eQTL for DHFR in artery (p = 6.7 × 10^–22^) and nerve (p = 5.9 × 10^–19^) but the association is only weak in whole blood (p = 1.3 × 10^–8^ compared to 2.8 × 10^–63^ for MSH3). The minor allele at this SNP is associated with an earlier age at onset of HD (p = 5.46 × 10^–10^)^38^ and increasing somatic instability in HD and DM1^32^.

The rs150393409 SNP is located within *FAN1*, a DNA repair gene that encodes a nuclease FAN1 that protects against expansion in the FXD mouse^53,54^. This SNP results in the substitution of Arg for His at amino acid 507 in FAN1, a change predicted to be deleterious or damaging in SIFT and PolyPhen, respectively. The directionality of the observed effect of the rs150393409 SNP would be consistent with FAN1 normally protecting against repeat expansion in women with the PM as well. Thus, although studies of larger cohorts are needed, our data suggest that genetic factors that affect somatic expansion in women with the PM are consistent with data from a mouse model of the FXDs and with other Repeat Expansion Diseases. This similarity between humans and mice with respect to the genetic factors involved in somatic expansion supports the idea that the FXD mouse model can provide useful insights into the expansion process in human PM carriers. The fact that the same SNPs are associated with disease risk in other Repeat Expansion Diseases lends weight to the idea that these diseases share a common underlying mutational mechanism.

It is notable that expansion can be readily detected in the blood of many PM human carriers. In an FXD mouse model, blood shows much less expansion than the brain^48^. A similar difference between the extent of expansion in blood and brain has been reported in other Repeat Expansion Diseases^55–58^. Thus, in PM carriers where expansion can be detected in blood, the extent of expansion in the brain maybe even larger. Since there is a direct relationship between repeat number and FXTAS age of onset^39^, this raises the possibility that the propensity to undergo somatic expansion could contribute to the variable penetrance of FXTAS pathology seen in PM carriers. Furthermore, since in the FXD mouse model the same genetic factors that affect expansion risk in somatic cells affect expansion in the germline, the genetic factors identified in this study as potential modifiers of somatic expansion risk, may also be modifiers of intergenerational expansion risk. These factors may account for some of the variances in expansion risk that are not explained by repeat number or the number of AGG interruptions^14^. Thus, a better understanding of the full range of genetic factors affecting expansion risk may contribute to better assessments of disease risk in PM carriers as well as the risk of transmission of FXS.

## Supplementary Information

Supplementary Information.

## Data Availability

Data and results generated from this project will be fully available from corresponding author upon request. Biological samples from subjects included in this study will be available under MTA agreement accordingly to the University of California, Davis policy.
