# Supplementary material for: Both cis and trans-acting genetic factors drive somatic instability in female carriers of the FMR1 premutation
Source: Sci Rep. 2022 Jun 21;12:10419. doi: 10.1038/s41598-022-14183-0 (PMC9213438; doi:10.1038/s41598-022-14183-0)
Supplement: Supplementary file 1 — Supplementary Information. [file 41598_2022_14183_MOESM1_ESM.docx]

**Supplementary Material**

**Supplementary Table 1:**Molecular measures, age at the initial visit (IV) and the final visit (FV) in the 24 cases of female carriers with multiple blood draws.

**
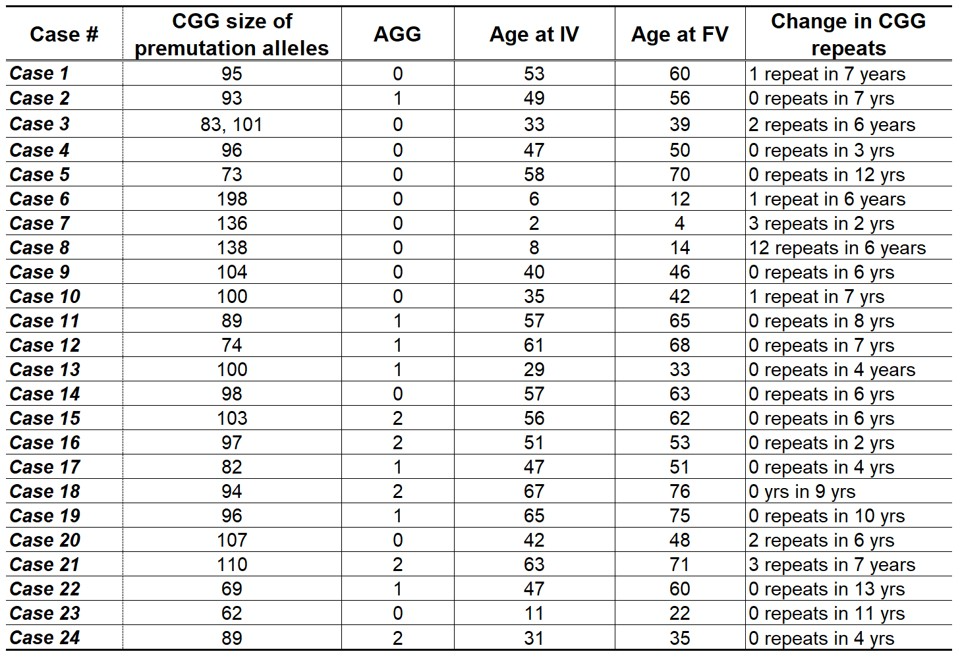
**
